# Acute and long-term outcomes of SARS-CoV-2 infection in school-aged children in England: Study protocol for the joint analysis of the COVID-19 schools infection survey (SIS) and the COVID-19 mapping and mitigation in schools (CoMMinS) study

**DOI:** 10.1371/journal.pone.0303892

**Published:** 2024-05-22

**Authors:** Katharine J. Looker, Elliot McClenaghan, Alison Judd, Livia Pierotti, Harriet Downing, Jody Phelan, Charlotte Warren-Gash, Kalu Ngozi, Fiona Dawe, Caroline Relton, Hannah Christensen, Alastair D. Hay, Punam Mangtani, Patrick Nguipdop-Djomo, Rachel Denholm

**Affiliations:** 1 Population Health Sciences, University of Bristol, Bristol, United Kingdom; 2 Centre for Academic Primary Care, University of Bristol, Bristol, United Kingdom; 3 Department of Infectious Disease Epidemiology, Faculty of Epidemiology and Population Health, London School of Hygiene & Tropical Medicine, London, United Kingdom; 4 Office for National Statistics, Newport, South Wales, United Kingdom; 5 NIHR Bristol Biomedical Research Centre, University of Bristol, Bristol, United Kingdom; 6 Department of Infection Biology, Faculty of Infectious and Tropical Diseases, LSHTM, London, United Kingdom; 7 Department of Non-Communicable Disease Epidemiology, Faculty of Epidemiology and Population Health, LSHTM, United Kingdom; 8 MRC Integrative Epidemiology Unit, University of Bristol, Bristol, United Kingdom; 9 Faculty of Epidemiology and Population Health, LSHTM, United Kingdom; 10 Health Data Research UK (HDR UK) South-West, Bristol, United Kingdom; Kyung Hee University School of Medicine, REPUBLIC OF KOREA

## Abstract

**Background:**

The symptom profiles of acute SARS-CoV-2 infection and long-COVID in children and young people (CYP), risk factors, and associated healthcare needs, are poorly defined. The Schools Infection Survey 1 (SIS-1) was a nationwide study of SARS-CoV-2 infection in primary and secondary schools in England during the 2020/21 school year. The Covid-19 Mapping and Mitigation in Schools (CoMMinS) study was conducted in schools in the Bristol area over a similar period. Both studies conducted testing to identify current and previous SARS-CoV-2 infection, and recorded symptoms and school attendance. These research data have been linked to routine electronic health record (EHR) data.

**Aims:**

To better understand the short- and long-term consequences of SARS-CoV-2 infection, and their risk factors, in CYP.

**Methods:**

Retrospective cohort and nested case-control analyses will be conducted for SIS-1 and CoMMinS data linked to EHR data for the association between (1) acute symptomatic SARS-CoV-2 infection and risk factors; (2) SARS-CoV-2 infection and long-term effects on health: (a) persistent symptoms; (b) any new diagnosis; (c) a new prescription in primary care; (d) health service attendance; (e) a high rate of school absence.

**Results:**

Our study will improve understanding of long-COVID in CYP by characterising the trajectory of long-COVID in CYP in terms of things like symptoms and diagnoses of conditions. The research will inform which groups of CYP are more likely to get acute- and long-term outcomes of SARS-CoV-2 infection, and patterns of related healthcare-seeking behaviour, relevant for healthcare service planning. Digested information will be produced for affected families, doctors, schools, and the public, as appropriate.

**Conclusion:**

Linked SIS-1 and CoMMinS data represent a unique and rich resource for understanding the impact of SARS-CoV-2 infection on children’s health, benefiting from enhanced SARS-CoV-2 testing and ability to assess a wide range of outcomes.

## Introduction

Children and young people (CYP) are an often-forgotten group in research into SARS-CoV-2 infection (which causes ‘COVID-19’ symptoms) [1]. It has been established that most infections in this age group present either without acute symptoms (asymptomatic) or with mild acute symptoms, for those SARS-CoV-2 variants that have been studied to date at least [2–4], although a small but important number of CYP in the early part of the pandemic developed severe symptoms that required hospitalisation, and in rare instances, paediatric inflammatory multisystem syndrome temporally associated with SARS-CoV-2 (PIMS-TS) [5]. There is a general lack of research characterising the symptom profile of acute SARS-CoV-2 infection in CYP, which is different to that of adults [4, 6]. The risk factors for acute symptoms have also not been well characterised, with most studies limited to severely ill, hospitalised children [7]. Uptake of vaccination among CYP is relatively low (as of July 2022, 62% of 12-15-year-olds and 81% of 16-17-year-olds had at least one dose; 45% and 70% respectively had two doses [8]). Understanding the acute symptom profile of SARS-CoV-2 infection is vital due to the emergence of new variants of concern (VOC) capable of escaping existing immunity, whether vaccine-induced or naturally-derived immunity, to cause re-infections.

Furthermore, it remains crucial to understand whether those CYP with acute symptomatic infection are at higher risk of subsequent long-term health effects, referred to as long-COVID. Estimates of the percentage of CYP with long-COVID following infection range from 3–14% [1, 4, 9, 10]. Previous studies have tended to only include CYP with a diagnosed/symptomatic infection, which may unavoidably bias estimates of the risk of long-COVID [1]. Diagnosis of long-COVID is difficult as current definitions are very broad. The National Institute for Health and Care Excellence (NICE) defines long-COVID as a collective term for ‘ongoing symptomatic COVID-19’ (signs and symptoms 4–12 weeks after infection) and ‘post-COVID-19 syndrome’ (signs and symptoms >12 weeks after infection), including in CYP [11]. The World Health Organization (WHO) has produced a clinical case definition of ‘post COVID-19 condition’ by a Delphi consensus for adults [12] while a research definition has been proposed for CYP [13]. Hereon we refer to long-COVID to mean ongoing symptoms >4 weeks after infection, in line with NICE’s collective definition. Looking at a wider range of outcomes, not limited to symptoms and long-COVID diagnoses, would help inform understanding of the long-term effects of SARS-CoV-2 infection in CYP.

Those CYP with long-COVID who are seen in primary care may represent only the tip of the iceberg meaning there may be substantial unmet need for care, but this has not been quantified. The characteristics of those CYP with persistent symptoms following a SARS-CoV-2 infection who do not present to primary care are not well understood. CYP not seen in primary care may have milder symptoms, or conversely, may progress to having more serious complications, presenting to A&E and/or requiring hospital admission, which may have been avoided by earlier intervention in primary care.

Further research into the risk, characterisation and risk factors of symptomatic infection–both acute and long-term–would provide deeper understanding of which CYP are most at risk of developing more severe clinical sequelae, and inform clinical management and service planning.

The Schools Infection Survey 1 (SIS-1) [14] in England (conducted by the London School of Hygiene & Tropical Medicine [LSHTM], in conjunction with the Office for National Statistics [ONS], the Department of Education, the Department of Health & Social Care and UK Health Security Agency [UKHSA]) ran over the school year 2020–21. The Bristol-based Covid-19 Mapping and Mitigation in Schools (CoMMinS) study [15] (led by the University of Bristol in partnership with Bristol City Council and UKHSA) ran from November 2020 to December 2021. Both surveys regularly tested primary and secondary school children for SARS-CoV-2 infection and antibodies and asked about school absence and recent symptoms experienced, with SIS including questionnaires specifically on ongoing symptoms. This means they were able to identify infections that would not otherwise have been captured by electronic health record systems, and offer a unique opportunity both for studying the symptom profile of all symptomatic acute infections, including those CYP with only mild symptoms, and for investigating the long-term effects of infection following asymptomatic and mild infections, as well as following clinically-apparent presentations.

SIS-1 data have been enhanced through linkage with electronic health record (EHR) data, specifically Hospital data (Hospital Episode Statistics [HES] data: Outpatients, Critical Care, Accident and Emergency, and Admitted Patient Care), Emergency Care Data Set (ECDS) data (a newer dataset which will replace the Accident and Emergency dataset), COVID-19 testing data (COVID-19 Second Generation Surveillance Systems [SGSS] data) and prescription data (Medicines dispensed in Primary Care NHS Business Services Authority [NHSBSA] data). Linkage with GP data (General Practice Extraction Service [GPES] Data for Pandemic Planning and Research [GDPPR] data) is planned. CoMMinS data have been linked with a local linked data resource, the Bristol, North Somerset & South Gloucestershire (BNSSG) SystemWide Dataset, which includes data from general practices, the Secondary Uses Service (SUS) dataset for North Bristol NHS Trust and University Hospitals Bristol and Weston NHS Foundation Trust, and SGSS SARS-CoV-2 positive infection data [16]. See Governance section (S1 File) for further details.

## Materials and methods

### Aims

Research Question (RQ) 1: What are the characteristics and risk factors of acute symptomatic SARS-CoV-2 infection in school-aged children in England?

1. Describe the acute symptom profile of SARS-CoV-2 infection
2. Investigate the predisposing sociodemographic and clinical factors (including VOC) associated with a higher risk of acute symptomatic SARS-CoV-2 infection (versus asymptomatic SARS-CoV-2 infection)
3. Explore patterns of clustering of acute symptoms (and compared with a control group with no history of SARS-CoV-2 infection).
  RQ2: What are the characteristics and risk factors of long-COVID in school-aged children in England?
4. Examine whether SARS-CoV-2 infection (versus no SARS-CoV-2 infection) is associated with a higher risk of (a) survey-reported persistent recent and long-COVID symptoms; (b) any (new) diagnosis of a condition; (c) any (new) pharmacological product prescribed in primary care; (d) health service attendance: (i) primary care visit, (ii) Accident and Emergency (A&E) visit, (iii) hospital admission, (iv) outpatient visit; (e) school absence due to illness. Timeframes for (1) ongoing symptomatic COVID-19 (4–12 weeks since infection) and (2) post-COVID-19 syndrome (more than 12 weeks since infection) will be examined, as these may have different clinical presentations.
5. Investigate the predisposing sociodemographic and clinical factors associated with a higher risk of persistent recent and long-COVID symptoms, incident diagnoses of conditions, prescriptions, health service attendance, and school absence, following a SARS-CoV-2 infection
6. Explore patterns of clustering of persistent recent and long-COVID symptoms and diagnoses of conditions (and compared with a control group with no history of SARS-CoV-2 infection).

### Study design

Retrospective observational analyses, using SIS-1 and CoMMinS data, will be undertaken to investigate the association between:

RQ1 (nested case-control study): *Acute symptomatic SARS-CoV-2 infection* (cases; versus asymptomatic SARS-CoV-2 infection [controls]) and *risk factors*
RQ2 (cohort study): *SARS-CoV-2 infection* (exposure; versus no infection) and *long-term outcomes*.

Follow-up in the surveys covered the end of SIS-1 (July 2021) and CoMMinS (December 2021). Follow-up in EHRs will cover 1^st^ April 2019-31^st^ March 2023 for SIS-1 participants and 1^st^ January 2019-31^st^ December 2021 for CoMMinS participants.

### Inclusion criteria

The inclusion criteria for the analyses are as follows:

- Enrolled and participated in one of the two school infection surveys (SIS-1 or CoMMinS)
- Age 4–18 years at enrolment into either school infection survey
- Provided at least one sample for SARS-CoV-2 testing.

### Definition of cases (RQ1) and exposure (RQ2)

For RQ1, cases will be defined as those with SARS-CoV-2 infection (Table 1) and at least one acute symptom recorded in questionnaires in the same interval as the infection (S1 Table), with controls being those with asymptomatic SARS-CoV-2 infection.

**Table 1:** Definition of a (first) SARS-CoV-2 infection in CoMMinS versus SIS-1.

	CoMMinS	SIS-1
**Survey data**	Date of:• a positive research SARS-CoV-2 infection PCR test • SARS-CoV-2 anti-NP antibody conversion from negative to positive^1^ • a positive SARS-CoV-2 anti-NP antibody assay^1^ • a self-reported SARS-CoV-2 infection	Date of:• a positive research SARS-CoV-2 infection PCR test • SARS-CoV-2 anti-NP antibody conversion from negative to positive^1^ • a positive SARS-CoV-2 anti-NP antibody assay^1^ • a self-reported SARS-CoV-2 infection
**Linked EHR data**	Date of: • BNSSG SWD primary care attributes data table: a confirmed SARS-CoV-2 infection or SARS-CoV-2 diagnosis code • SGSS: a positive SARS-CoV-2 infection PCR test • SUS: a confirmed SARS-CoV-2 infection or SARS-CoV-2 diagnosis code	Date of: • GDPPR: a confirmed SARS-CoV-2 infection or SARS-CoV-2 diagnosis code • SGSS: a positive SARS-CoV-2 infection PCR test • HES: a confirmed SARS-CoV-2 infection or SARS-CoV-2 diagnosis code

PCR: Polymerase Chain Reaction; anti-NP: anti-nucleoprotein; BNSSG SWD: Bristol, North Somerset, and South Gloucestershire System Wide Dataset; SGSS: Second Generation Surveillance System; GDPPR: COVID-19 General Practice Extraction Service (GPES) Data for Pandemic Planning and Research. ^1^Only if prior to first vaccine dose, i.e., reflecting natural infection rather than vaccine-derived immunity.

For RQ2, exposure will be defined as those with SARS-CoV-2 infection (Table 1), with the unexposed group being those without SARS-CoV-2 infection. Follow-up for unexposed CYP will end on first SARS-CoV-2 infection, after which time they will become part of the exposed group.

A positive antigen test, positive antibody test, documented seroconversion or SARS-CoV-2 diagnosis will be used to define SARS-CoV-2 infection, in order to capture infection as accurately as possible (Table 1). Only first SARS-CoV-2 infections will be considered in initial analyses.

S1 Table lists acute (‘recent’) symptom data collected by SIS-1 and CoMMinS, and how these have been harmonised (relevant for definition of cases for RQ1). In short, SIS-1 participants were asked to report specific symptoms in the 7 days before or after sampling (‘recent’ symptoms) in each data collection round. CoMMinS participants were asked if they experienced specific symptoms in the past 30 days (‘recent’ symptoms) in each data collection round. EHR data will be used to supplement survey data on symptoms.

### Risk factors (RQ1)/covariates (RQ2)

Survey (SIS-1/CoMMinS) data on a range of risk factors/covariates are available, including age, gender, ethnicity, deprivation, symptomatic acute SARS-CoV-2 infection, SARS-CoV-2 vaccination, school type, geographical location, asthma, diabetes, heart condition, other long-term medical condition, and hospital admission in previous 12 months. EHR data also include information on a similar range of risk factors/covariates, with more detail on pre-existing medical conditions. Where EHR data are used to define a risk factor/covariate, the most recent data prior to index date will be used. S2 Table describes the data type, definition and data sources for the risk factors/covariates. S1 Table lists how the risk factor/covariate data for SIS-1 and CoMMinS have been harmonised.

### Outcomes (RQ2)

The analyses for RQ2 will consider self-reported recent symptoms that are ‘persistent’ (repeated) for (1) 4–12 weeks and (2) >12 weeks (see Definition of outcomes (RQ2) section in S1 File and S3 Table for more details), and (self-reported) school absences that are reported to be due to illness (SARS-CoV-2- or non-SARS-CoV-2-related), using SIS-1 and CoMMinS data. Clinical diagnoses of conditions, prescriptions and health service attendance occurring (1) up to 12 weeks and (2) after 12 weeks following (first) SARS-CoV-2 infection will be examined. These timeframes were selected to be in line with NICE’s definitions for ongoing symptomatic COVID-19 and post-COVID-19 syndrome: these separate definitions may have different clinical presentations. If there are multiple records of the same diagnosis, prescription or health service attendance, the earliest will be used in the first instance. However, multiple prescriptions for the same product, and multiple health service attendances, will also be investigated.

The analyses will use a broad approach of investigating the association between SARS-CoV-2 infection and several chapters from the 10^th^ revision (2019 version) of the International Statistical Classification of Diseases and Related Health Problems (ICD-10) [17], and the corresponding chapters of the British National Formulary for Children (BNFC) [18] that relate to these, rather than for just a limited few diagnoses and prescriptions or just a long-COVID diagnosis on its own. This will enable wider assessment of the long-term effects of infection, and avoid assumptions about the nature of long-COVID in CYP insofar as the ICD-10/BNFC chapters considered are those that have most clinical relevance for CYP and their families (see Previous patient and public involvement (PPI) section in S1 File for more details) [19].

### Data analysis

Initial descriptive statistics will be used to describe the demographic and clinical characteristics of the participants in the two cohorts at the start of the surveys. Missing data patterns will be explored, and missing values will be imputed using multiple imputation where appropriate.

Mixed-effects logistic regression models with a random effect for school will be used to estimate odds ratios (ORs) for the association between symptomatic SARS-CoV-2 infection and risk factors (RQ1), and for the association between SARS-CoV-2 infection, and persistent/long-COVID symptoms and school absence, split by occurring over 4–12 weeks and over >12 weeks (RQ2).

Mixed-effects Cox regression models accounting for clustering within schools will be used to calculate hazard ratios (HRs) for the association between SARS-CoV-2 infection, and diagnoses of conditions, prescriptions and health service attendance (RQ2). Follow-up will be censored at the first of: outcome; death; end of follow-up. CYP may have multiple reports of SARS-CoV-2 infection. As a first step, patterns of infection will be described, and different definitions for exposure may be considered.

Unadjusted and adjusted effect estimates will be calculated. Subgroup/stratified analyses will be conducted for selected (important) risk factors/covariates including primary care attendance, and calendar time, e.g., events occurring (a) during SIS-1 and CoMMinS (correlating with regular testing and symptom questionnaires); and (b) after SIS-1 and CoMMinS (when SARS-CoV-2 infection is based solely on healthcare records). Demographic and clinical characteristics of participants who do and do not attend primary care will be compared, and regression models will also be used to investigate the probability of a participant with a specific symptom attending primary care. Planned sensitivity analyses include: repeat analyses but excluding those symptoms that are non-specific (e.g., headache, fever); alternative definitions of symptomatic versus asymptomatic infection (RQ1).

Symptom burden and combinations of symptoms will be analysed and presented in a heat map/upset plot. Exploratory latent class analysis (LCA) may be conducted to classify the various symptoms into latent classes (groups) including by strata of interest if the results of the previous analyses indicate that these cluster differently in different individuals, in order to define distinct clinical presentations. Prescriptions, school absence and health service attendance will be compared between different groups. Results will be compared with those with no history of SARS-CoV-2 infection.

Data for SIS-1 will be analysed separately to those for CoMMinS. Effect estimates from SIS-1 and CoMMinS will then be pooled using fixed-effects meta-analysis.

It is expected that the analyses will commence in November 2023. All data analyses will be done using the R programming language. Study reporting will follow STROBE guidelines [20].

### Sample size

In SIS-1, 14,842 students participated in at least one study round [21], while in CoMMinS, a sample size of ~2,000 participants (~9,000–10,000 saliva samples) was achieved [15].

Table 2 shows example (simplified, i.e., without accounting for clustering within schools) power calculations for various effect (odds ratio) sizes combined with (1) various percentages with the risk factor (exposure) (RQ1); and (2) various outcome risks in the unexposed group (RQ2), using SIS-1 as an example, using the *power twoprop* command in Stata 17.0.

**Table 2:** Example power calculations based on SIS-1 (using N = 14,800 CYP).

Odds ratio	RQ1: Acute symptomatic SARS-CoV-2 infection (cases; versus asymptomatic SARS-CoV-2 infection [controls]) and risk factors (exposure)^1^		RQ2: SARS-CoV-2 infection (exposure; versus no infection) and long-term outcomes^2^	
	Percentage with risk factor in control group	Estimated power	Outcome risk in unexposed group	Estimated power
**2.0**	10%	68.1%	1%	93.7%
	15%	80.2%	5%	100%
	20%	86.7%	10%	100%
**1.5**	10%	27.7%	1%	43.1%
	15%	35.5%	5%	98.8%
	20%	41.5%	10%	100%

All calculations assume 95% significance level. ^1^Assuming an approximate control to case ratio of 2:1 and 150 symptomatic cases; ^2^Assuming an overall sample size of 14,800 and an estimated infection rate of 15%.

### Ethics statement

UKHSA and LSHTM Research Ethics Committees (RECs) gave ethical approval for the original study for SIS-1. Informed consent for the original SIS-1 study was sought from parents/guardians for children under 16 years, with parents/guardians asked to involve their children in the decision process by explaining why the study was being undertaken and how their children could help by taking part. Informed consent for those children aged 16 years and over was sought from the students themselves. An electronic link was provided to give information about the study, along with the online consent form and a short online questionnaire for enrolment. Enrolment was not offered to secondary school students who were considered by school staff as being not competent to provide informed consent.

Ethical approval for the SIS-1 data linkage (covering the period covering 1^st^ April 2019-31^st^ March 2023 for EHR data) was received from the Confidential Advisory Group (22/CAG/0130) and East of England–Cambridge South Research Ethics Committee (23/EE/0013). Re-consent for the data linkage was not sought from SIS-1 participants. ONS considered that obtaining re-consent would be a significant administrative undertaking requiring their staff to access the (identifiable) consent forms and contact details for all participants. This degree of interaction with participant data would have been far in excess of the interaction required by the study: a largely automated linkage process with only de-identified data available for analysis. Instead, an updated privacy notice was published on the SIS-1 information page of ONS’s website (also available at https://elucidatestudy.blogs.bristol.ac.uk/use-of-study-data/). SIS-1 participants were informed about the study through an email update to those schools which participated in SIS-1 and given a notice period to opt out.

Access to the data for analysis will be restricted to employees of the University of Bristol and LSHTM who are named ONS-accredited researchers. They are permitted to access de-identified data only, and will do so via ONS’s secure data environment. Only aggregated outputs will be taken out of the environment, after undergoing data minimisation and disclosive risk checks.

Collection of CoMMinS research data received the required ethical approval from London–City & East Research Ethics Committee (REC reference 20/HRA/4876). Online consent was sought from parents/guardians for children aged under 16 years. Children of secondary school age and above were simultaneously asked to assent. Consent was obtained directly from the student themselves if aged 16 years or over. Students who were not, in the judgement of their headteacher and/or their parent/guardian, able to participate in the assent/consent process, were excluded. NHS Governance for the CoMMinS linkage went through the BNSSG Integrated Care Board’s governance structure. Consent for the data linkage (covering the period 1^st^ January 2019-31^st^ December 2021 for EHR data) was sought in the original online consent forms.

As for SIS-1, only de-identified data will be analysed. Access to the data for analysis will be restricted to University of Bristol CoMMinS researchers. Only aggregated outputs will be exported, after appropriate data minimisation and disclosive risk checks.

### Patient and public (PPI) involvement

Patients and the public will be engaged throughout the study. Six-monthly meetings will be held with parents/guardians, and quarterly meetings with young people, aiming for an average of eight participants at each meeting. Diversity in participant geographical area of residence and ethnicity will be increased by e.g., recruiting directly from participating SIS-1/CoMMinS schools and from underserved communities via established links between the University of Bristol and communities. Summary reports of the PPI meetings will be published on the study-specific website https://bristol.ac.uk/elucidate-study. Two parents/guardians from families affected by long-COVID will be invited to join the group of advisors providing input on the study (see Governance section in S1 File for more information about the advisors) to ensure PPI is embedded at all levels and in all key decisions. Appropriate training for PPI involvement will be provided. PPI activities, training and impact will be continuously reviewed and evaluated.

## Discussion

### Benefits

SIS-1 and CoMMinS were able to capture SARS-CoV-2 infections which would otherwise have been missed through routine testing of (only) symptomatic CYP [2–4]. Although the UK Government did introduce a policy of twice-weekly lateral flow testing for schools, this was only for secondary school pupils, and only introduced in March 2021. SIS-1 and CoMMinS therefore offer a unique opportunity for studying the acute- and long-term effects of infections which would not have been identified outside of a study setting. This study is one of the few to have linked research data with medical record data, which will greatly enrich the analyses, particularly in characterising the trajectory of long-COVID in CYP, and understanding the extent of the potential unmet need for care among those who do not access healthcare services. The study will also benefit from long follow-up relative to previous studies, suitable control groups, and full information on covariates such as co-morbidities.

### Limitations

This study has some potential limitations. First, there is limited follow-up in the research data for both SIS-1 and CoMMinS. Not all of those recruited will have given samples and completed questionnaires at each study interval, meaning some SARS-CoV-2 infections will be missed. In SIS-1, 72% of students had at least 2 completed visits (including enrolment), but only 17% attended rounds 1, 2, 4 and 6 (considered to the most realistically attainable level of follow-up) [22]. In CoMMinS, 98%, 91% and 93% of primary school, secondary school and sixth form students, respectively, had at least 2 completed visits, compared to only 50%, 15% and 6% for 6 or more completed visits (Pierotti, L., manuscript in preparation). However, the use of research data on infections means many more infections will have been identified than would otherwise, as noted above, while antibody and SGSS data will be used alongside the survey data on current infections to identify as many infections as possible for those time intervals where survey data are not available.

Second, and relatedly, some symptoms may also be missed through loss-to-follow-up, while additionally, the symptoms surveyed are not exhaustive and do not have a given definition, and therefore may not adequately capture the morbidity associated with SARS-CoV-2 infection. In both SIS-1 and CoMMinS, participants were able to select ‘Other’ symptom and give details in a free-text box, but this requires a greater level of active participation to record a symptom than simply selecting a check box, while categorising and coding free-text answers present their own challenges.

Third, the study will use data on repeated recent symptoms from both SIS-1 and CoMMinS as a proxy for persistent symptoms. It is assumed that if a symptom is recorded twice for a particular individual within a certain timeframe, then that symptom is persistent. If this is not the case, this may lead to over-estimation of the association of infection with persistent symptoms. Moreover, SIS-1 and CoMMinS did not record recent symptoms in the same way: SIS-1 asked about symptoms within 7 days of testing, while CoMMinS asked about symptoms in the past 30 days. However, SIS-1 data collected specifically on ongoing, long-COVID symptoms will be analysed alongside data on repeated recent symptoms and the consistency of the results compared.

Fourth, it is challenging to define symptomatic versus asymptomatic infection (RQ1) in the context of a large range of possible symptoms and associated severity and duration, and set against a multitude of other circulating infections which often have similar symptoms. Planned sensitivity analyses will however test the strength of case and control definitions.

Fifth, SIS-1 and CoMMinS took place during the circulation of wild-type and delta-type SARS-CoV-2 variants. Due to the historic nature of the surveys, research data on more recent variants will not be available for analysis. However, it is important to understand the dynamics of earlier variants as there are implications for current infections, and especially for the long-term health of CYP who are still affected by infection earlier on in the pandemic [10].

Lastly, long-term outcomes may not be adequately captured by electronic health records, as these are restricted to coded diagnoses of conditions, prescriptions and health service attendances, while some affected children may not present to healthcare at all. However, the ability of this study to analyse a wide range of possible outcomes not limited to symptoms means that the plausibility and impact of single associations can be assessed by analysing the consistency of associations across different outcomes. Furthermore, the inclusion of active SARS-CoV-2 testing means that this study will be able to evaluate the potential unmet need for care and barriers to accessing care, through quantifying the numbers with symptoms who do not attend primary care and the broad characteristics of these individuals, respectively.

S4 Table lists anticipated challenges/limitations in full, and the steps that will be taken to minimise/mitigate the effects of these See also Previous patient and public involvement (PPI) section in S1 File for details on how PPI input informed these [19].

### Impact

This project will inform and support understanding and clinical management of acute symptomatic SARS-CoV-2 infection and long-COVID in CYP by:

1. Characterising the clinical trajectory of long-COVID in CYP
2. Identifying which groups of CYP are most at risk of short- and long-term effects of SARS-CoV-2 infection
3. Informing the extent to which CYP with long-COVID attend primary care (and hence the potential unmet need for care), and the influencing sociodemographic and clinical characteristics
4. Producing appropriate digested information and messaging for the support of CYP and their families.

Study findings will be published in academic journals and policy reports, and presented at relevant conferences. Clinical networks, blogs, and ARC BITEs (Applied Research Collaboration Brokering Innovation Through Evidence) will be utilised to disseminate information to clinicians, with the view to findings ultimately being incorporated into clinical guidance via NICE. Information sheets and a patient video (translated into different languages) will be produced, and made available online, for affected children and their families. These outputs will be created in collaboration with our PPI contributors, the National Institute for Health and Care Research (NIHR), the UKHSA, and the University of Bristol Impact Development and Public Engagement teams. A joint media strategy will be devised through consultation with the media offices of the University of Bristol, LSHTM and ONS. UKHSA will be consulted to produce public health messaging as appropriate. The findings will be shared on the project website, which is available at https://bristol.ac.uk/elucidate-study, and on the CoMMinS website https://commins.org.uk/. The PPI contributors will be engaged at regular intervals to give input and feedback such as on draft manuscripts and policy reports.

## Supporting information

S1 File Supporting information. (PDF)

S1 Table Comparison of key demographic, clinical, symptom and educational variables for SIS-1 and CoMMinS, and result of harmonization of these variables for analysis. (PDF)

S2 Table Analysis risk factors (RQ1)/covariates (RQ2), description and informing data sources. (PDF)

S3 Table Definition of ongoing symptomatic COVID-19 and post-COVID-19 syndrome using self-reported symptom data from CoMMinS versus SIS. (PDF)

S4 Table Expected challenges/limitations, and steps that will be taken to minimise/mitigate effect. (PDF)
