# Supplementary material for: Acute and long-term outcomes of SARS-CoV-2 infection in school-aged children in England: Study protocol for the joint analysis of the COVID-19 schools infection survey (SIS) and the COVID-19 mapping and mitigation in schools (CoMMinS) study
Source: PLoS One. 2024 May 22;19(5):e0303892. doi: 10.1371/journal.pone.0303892 (PMC11111005; doi:10.1371/journal.pone.0303892)
Supplement: S1 File — (PDF) [file pone.0303892.s001.pdf]

Supporting information

Acute and long-term outcomes of SARS-CoV-2 infection in school-aged children in England: Study protocol for the joint analysis of the COVID-19 Schools Infection Survey (SIS) and the COVID-19 Mapping and Mitigation in Schools (CoMMinS) Study

KJ Looker, E McClenaghan, A Judd, L Pierotti, H Downing, J Phelan, C Warren-Gash, K Ngozi, F Dawe, C Relton, H Christensen, AD Hay, P Mangtani, P Nguipdop-Djomo and R Denholm

Governance

The research team will consist of Principal Investigators, Co-Investigators and collaborators, and will meet regularly to discuss progress of the analyses. A group of advisors will provide input on the methods and results, and link in with appropriate networks for dissemination. Meetings with the advisors will take place at six-monthly intervals. Patient and public involvement (PPI) participants will be fully involved in decisions throughout.

The application to process SIS-1 data linked to electronic health record (EHR) data was submitted under Article 6.1.(e) of the UK GDPR. The request for processing special categories of personal data was submitted under Article 9.2.(j) of the UK GDPR. The University of Bristol and the London School of Hygiene & Tropical Medicine (LSHTM) are joint data controllers for the study. The University of Bristol, LSHTM and the Office for National Statistics (ONS) are joint data processors for the study. One data cut from NHS England was requested covering 1st April 2019-31st March 2023. Section 251 support was requested for the transfer of personal data (NHS number and date of birth for SIS-1 participants, along with a unique study identifier) from ONS to NHS England to enable NHS England to generate this data cut.

SIS-1 data are stored at ONS. ONS has strict security procedures that follow government standards. The University of Bristol and LSHTM researchers will not see any personal data. Only de-identified data will be available for analysis, within ONS's Secure Research Service (SRS). The level of data in the outputs will be aggregate data with small numbers suppressed, compliant with all disclosure conditions of all the relevant datasets used.

The linkage of electronic health records to CoMMinS data has been done entirely separately, through the Bristol, North Somerset, and South Gloucestershire (BNSSG) Integrated Care Board (ICB). The condition for processing is Article 6.1.(e) of the UK GDPR. The condition for processing of special category personal data is Article 9.2.(h) of the UK GDPR. The University of Bristol is processing the linked data on behalf of the data controller BNSSG ICB. EHR data covering the period 1st January 2019-31st December 2021 were requested. The University of Bristol researchers will not see any personal data. They will access the de-identified data through a secure data safe haven.

Further details on the methods

Definition of outcomes (RQ2)

Self-reported persistent symptoms

S3 Table describes how persistent self-reported symptoms will be defined in the two surveys. For CoMMinS, binary measures will be used to detail whether a recent symptom persisted over approximately 4-12 weeks (correlating to ongoing symptomatic COVID-19) and more than 12 weeks (correlating to post-COVID-19 syndrome). For SIS-1, which additionally asked specifically about long-COVID at the end of the survey, binary measures will be used to detail whether a symptom persisted for approximately 4-12 weeks and more than 12 weeks separately for each of the two available data sources on symptoms: (a) derived using data on recent symptoms (as for CoMMinS) and (b) directly measured through the questions specifically on long-COVID (S3 Table).

School absences

CoMMinS participants were asked how many days off school they had had (or would have needed, even if schools were closed or in school holidays) in the past 30 days due to medically diagnosed SARS-CoV-2 (confirmed by a doctor), due to suspected SARS-CoV-2 (not medically confirmed), and due to other illness not related to SARS-CoV-2. These data will be combined for the analyses as number of days off school due to any illness (SARS-CoV-2- or non-SARS-CoV-2-related) in the past month for CoMMinS.

SIS-1 participants were asked whether they had been absent from school in the past 4 weeks for any reason, and if so, whether this was due to SARS-CoV-2, not due to SARS-CoV-2, or both. Where absences were due to SARS-CoV-2, participants were asked whether these were due to testing positive for SARS-CoV-2, having SARS-CoV-2 symptoms/suspected SARS-CoV-2 (but no positive test), being told to isolate due to a potential contact with a case of SARS-CoV-2, school being shut, quarantining due to travel outside of the UK, shielding, having long-term symptoms after being positive for SARS-CoV-2 in the past or other SARS-CoV-2-related reason. Where absences were not due to SARS-CoV-2, participants were asked whether these were due to (non-SARS-CoV-2-related) respiratory illness, vomiting/diarrhoea/nausea/abdominal pain, other illness, medical/dental/hospital appointment/care or other reason. Data on number of days off school for those participants where the reason for absence was any of: having SARS-CoV-2 symptoms/suspected SARS-CoV-2 (but no positive test), having long-term symptoms after being positive for SARS-CoV-2, (non-SARS-CoV-2-related) respiratory illness, vomiting/diarrhoea/nausea/abdominal pain, or other illness will be analysed as number of days off school due to illness (SARS-CoV-2 and non-SARS-CoV-2 illness) in the past month for SIS.

In both surveys, the number of days absent from school due to illness will be derived for each participant (S1 Table). Those that are in the top quarter of the distribution will be defined as having a high number of absences due to ill health in each survey.

Diagnoses and prescriptions

Clinical diagnoses within the ICD-10 chapters for respiratory, cardiovascular, gastrointestinal, psychological/psychiatric, and nervous system diseases, and signs and symptoms not elsewhere classified (which include fever, headache, fatigue and pain) (Box 1), and BNFC codes relating to these chapters, will be extracted.

Box 1: Selected ICD-10 chapters

V Mental and behavioural disorders
VI Diseases of the nervous system
IX Diseases of the circulatory system
X Diseases of the respiratory system
XI Diseases of the digestive system
XVIII Symptoms, signs and abnormal clinical and laboratory findings, not elsewhere classified

Health service attendance

Date of contact with/admission to either primary care, A&E, hospital as an inpatient, or hospital as an outpatient, will be extracted.

Previous patient and public involvement (PPI)

Prior to developing this protocol, views about long-COVID in children and young people (CYP) were gathered from young people, parents and doctors between 9th March and 30th April 2021 to inform the research questions and methods(1). This was done by:

- holding an online meeting with seven young people aged 13-18 years from the NIHR Bristol Biomedical Research Centre Young People's Advisory Group (YPAG);
- holding an online meeting with five families whose children, aged 10-16 years, have long-COVID or suspected long-COVID;
- sending out a survey that was subsequently completed by four GPs and one paediatrician, and holding an online meeting with two paediatricians.

The PPI contributors said that:

1. The SARS-CoV-2 pandemic is ongoing and accumulation of knowledge is still in the early stages.
2. Clinical understanding of long-COVID in CYP is currently extremely limited. Long-COVID in CYP is not well defined, and it may be difficult for doctors to distinguish between long-COVID and other conditions. It still needs to be understood whether long-COVID is a new condition in itself, or a group of conditions which are already known about, like post viral fatigue.
3. For these reasons, long-COVID is likely to have been under-diagnosed to some extent to date, although it is still unclear what exactly long-COVID in CYP means.
4. Symptoms attributed to long-COVID varied between the families spoken to, and the symptoms their children had experienced were more wide-ranging than those listed on the NHS website(2) at the time of the meeting. Not all had been tested at the time of their infection.
5. The families whose children have long-COVID or suspected long-COVID ranked extreme tiredness, shortness of breath, chest pain, heart palpitations, depression, anxiety, feeling sick or stomach pain, diarrhoea, headaches or fever, and allergies in the top 3 most harmful symptoms at least once.
6. Of the symptoms listed on the NHS website(2) for long-COVID at the time of the meetings, feeling sick or stomach pain, extreme tiredness, and headaches were the symptoms commonly ranked as most harmful by young people and by families whose children have long-COVID or suspected long-COVID.

From this it was concluded that:

1. Considering a wider range of symptoms, and looking more broadly at things like GP and hospital visits, and school attendance, might be a better way at this time of assessing how SARS-CoV-2 has affected CYP. It is important to be aware of things like the extent to which healthcare is accessed according to need. The impact on CYP with milder symptoms may be missed if only EHR data are analysed.
2. Feeling sick or stomach pain, extreme tiredness, and headaches will be important symptoms to consider in the analyses.

3. Some SARS-CoV-2 infections which occurred early in the pandemic will not have been recorded, affecting the measurement of long-term health outcomes (e.g., misclassification of exposure bias).

These findings and conclusions have directly influenced the diagnoses/symptoms that will be included in the analyses (i.e., taking a broad, exploratory approach), what data will be used to investigate the long-term adverse effects SARS-CoV-2 has on CYP to include both EHR and research data, and consideration of potential biases due to testing strategy and access to tests leading to misclassification of infections over time.

Supporting information references

1. Looker KJ, Bell M, Skerry R, Pike L, Lewis SJ, Relton CL, et al. https://commins.org.uk/documents/Long-COVID-in-children-report-21_07_21.pdf Long COVID in children: A report summarising the views of young people, parents and doctors. 2021.
2. Long-term effects of coronavirus (long COVID) https://www.nhs.uk/conditions/coronavirus-covid-19/long-term-effects-of-coronavirus-long-covid/ Accessed 23/09/21.
