# Supplementary material for: Acute and long-term outcomes of SARS-CoV-2 infection in school-aged children in England: Study protocol for the joint analysis of the COVID-19 schools infection survey (SIS) and the COVID-19 mapping and mitigation in schools (CoMMinS) study
Source: PLoS One. 2024 May 22;19(5):e0303892. doi: 10.1371/journal.pone.0303892 (PMC11111005; doi:10.1371/journal.pone.0303892)
Supplement: S1 Table — (PDF) [file pone.0303892.s002.pdf]

**S1 Table: Comparison of key demographic, clinical, symptom and educational variables for SIS-1 and CoMMinS, and result of harmonization of these variables for analysis.**

| Type of data | Analysis variable | CoMMinS | | | | | SIS | | | | | Harmonised | | |
|---|---|---|---|---|---|---|---|---|---|---|---|---|---|---|
| | | Description of informing survey variable(s) | Name(s) of informing survey variable(s) | Type of informing survey variable(s) | Time of data collection for informing survey variable(s) | Available categories for analysis across all informing survey variables | Description of informing survey variable(s) | Name(s) of informing survey variable(s) | Type of informing survey variable(s) | Time of data collection for informing | Available categories for analysis across all informing survey variables | Name of analysis variable | Type of analysis variable | Categories of analysis variable |
| Demographic | Gender | Gender | gender_id | N | B | Female<br>Male<br>Other<br>Prefer not to answer | Gender | enrol_gender | S | B | Female<br>Male<br>Other<br>Prefer not to say | gender | C | Female<br>Male<br>Other |
| | | | gender_other | F | B | | | | | | | | | |
| | Age | Week of birth | WoB | N | B | <Age in weeks> | Date of birth | enrol_dob | S | B | <Age in weeks> | age | N | <Age in years> |
| | | Month of birth | MoB | N | B | | | | | | | | | |
| | | Year of birth | YoB | N | B | | | | | | | | | |
| | Ethnicity | Ethnicity | ethnicity_cat1 | N | B | African<br>Caribbean<br>Any other Black / African / Caribbean background<br>Bangladeshi<br>Chinese<br>Indian<br>Pakistani<br>Any other Asian background<br>English / Welsh / Scottish / Northern Irish / British<br>Irish<br>Gypsy or Irish traveller | Ethnicity | enrol_ethnic_asia | S | B | Black / African / Caribbean / Black British - African<br>Black / African / Caribbean / Black British - Caribbean<br>Black / African / Caribbean / Black British - Any other Black / African / Caribbean<br>Asian / Asian British - Bangladeshi<br>Asian / Asian British - Chinese<br>Asian / Asian British - Indian<br>Asian / Asian British - Pakistani<br>Asian / Asian British - Any other Asian background | ethnicity | C | Black / African / Caribbean / Black British<br>Asian / Asian British<br>White<br>Other ethnic group<br>Mixed / Multiple ethnic groups |
| | | | ethnicity_cat2 | N | B | | | enrol_ethnic_blk | S | B | | | | |
| | | | ethnicity_cat2_other | F | B | | | enrol_ethnic_main | S | B | | | | |
| | | | | | | | | enrol_ethnic_mix | S | B | | | | |
| | | | | | | | | enrol_ethnic_other | S | B | | | | |
| | | | | | | | | enrol_ethnic_other_spec | S | B | | | | |
| | | | | | | | | enrol_ethnic_wht | S | B | | | | |

| | | | | | | | | | | | | | | |
|---|---|---|---|---|---|---|---|---|---|---|---|---|---|---|
| | | | | | | Any other White background<br>Arab<br>White and Asian<br>White and Black African<br>White and Black Caribbean<br>Any other mixed / multiple ethnic group<br>Any other ethnic group<br>Prefer not to say | | | | | White - English / Welsh / Scottish / Northern Irish / British<br>White - Irish<br>White - Gypsy or Irish Traveller<br>White - Any other White background<br>Other ethnic group - Arab<br>Mixed / Multiple ethnic groups - White and Asian<br>Mixed / Multiple ethnic groups - White and Black African<br>Mixed / Multiple ethnic groups - White and Black Caribbean<br>Any other mixed / multiple ethic background<br>Other ethnic group - Any other ethnic group | | | |
| | Socio-economic deprivation | Index of Multiple Deprivation 2019 (deciles) | lsoa11cd | T | B | 1 (most deprived)<br>2<br>3<br>4<br>5<br>6<br>7<br>8<br>9<br>10 (least deprived) | Index of Multiple Deprivation 2019 (deciles) | enrol_postcode | S | B | 1 (most deprived)<br>2<br>3<br>4<br>5<br>6<br>7<br>8<br>9<br>10 (least deprived) | Index of Multiple Deprivation 2019 (deciles) | C | 1 (most deprived)<br>2<br>3<br>4<br>5<br>6<br>7<br>8<br>9<br>10 (least deprived) |
| | School | School type | school_type | T | B | Primary<br>Secondary<br>Sixth Form | School type | enrol_schl_year<br>enrol_schl_year_info | S<br>S | B<br>B | Primary<br>Secondary<br>Sixth Form | school_type | C | Primary<br>Secondary<br>Sixth Form |
| Clinical | Long term medical condition | Has long term medical condition | health_status | N | F | Has a long term medical condition<br>Does not have a long term medical condition<br>Has asthma<br>Has chronic lung disease<br>Has heart disease<br>Has high blood pressure<br>Has diabetes<br>Has depression<br>Has anxiety<br>Has immunodeficiency<br>Has kidney disease<br>Has cancer<br>Has other long term medical condition | Has long term medical condition | ext_child_illness | S | B | Has a long term medical condition<br>Does not have a long term medical condition<br>Do not know if have a long term medical condition<br>Prefer not to say if have a long term medical condition<br>Has diabetes<br>Has asthma<br>Has heart condition<br>Has other long term medical condition | long_term_condition | Bi | Has a long term medical condition<br>Does not have a long term medical condition |
| | | | health_change_description | F | F | | | ext_child_illness_what | S | B | | | | |
| | | | long_term_medical_condition | N | B | | | ext_child_illness_heart | S | B | | asthma | Bi | Has asthma<br>Does not have asthma |
| | | | medical_condition__1 | N | B | | | ext_child_illness_oth | S | B | | | | |
| | | | medical_condition__2 | N | B | | | | | | | diabetes | Bi | Has diabetes<br>Does not have diabetes |
| | | | medical_condition__3 | N | B | | | | | | | | | |
| | | | medical_condition__4 | N | B | | | | | | | heart_condition | Bi | Has heart condition<br>Does not have heart condition |
| | | | medical_condition__5 | N | B | | | | | | | | | |
| | | | medical_condition__6 | N | B | | | | | | | | | |
| | | | medical_condition__7 | N | B | | | | | | | | | |
| | | | medical_condition__8 | N | B | | | | | | | | | |
| | | | medical_condition__9 | N | B | | | | | | | | | |
| | | | medical_condition__10 | N | B | | | | | | | | | |
| | | | medical_condition__11 | N | B | | | | | | | | | |
| | | | other_medical_condition | F | B | | | | | | | | | |

| | | | | | | | | | | | | | | |
|---|---|---|---|---|---|---|---|---|---|---|---|---|---|---|
| COVID-19 symptoms | Acute COVID-19 symptoms | No symptoms | no_symptoms | N | BF | Had no symptoms in last 30 days | Symptom +/- 7 days of sampling | ext_SYMPTOM / fw_SYMPTOM | S | BF | Had shortness of breath / difficulty breathing in 7 days before / after sampling<br>Had headaches in 7 days before / after sampling<br>Had muscle ache (myalgia) in 7 days before / after sampling<br>Had tiredness / fatigue in 7 days before / after sampling<br>Had nausea / vomiting in 7 days before / after sampling<br>Had abdominal pain in 7 days before / after sampling<br>Had diarrhoea in 7 days before / after sampling<br>Had loss or change to their sense of smell or taste in 7 days before / after sampling | recent_symptoms | Bi | Recent symptoms<br>No recent symptoms |
| | | Symptom in last 30 days | fever_1 | N | BF | Had fever in last 30 days | Date of onset | ext_SYMPTOM_date / fw_SYMPTOM_date | S | BF | | | | |
| | | | shivers_chills_1 | N | BF | Has shivers / chills in last 30 days | Duration | ext_SYMPTOM_duration / fw_SYMPTOM_duration | S | BF | | recent_fever | Bi | Recent fever and / or shivers<br>No recent fever and / or shivers |
| | | | cough_1 | N | BF | Had cough in last 30 days | | | | | | recent_cough | Bi | Recent cough<br>No recent cough |
| | | | runny_nose_1 | N | BF | Had runny nose in last 30 days | | | | | | recent_sneezing | Bi | Recent runny nose and / or sneezing<br>No recent runny nose and / or sneezing |
| | | | sneezing_1 | N | BF | Had sneezing in last 30 days | | | | | | recent_sore_throat | Bi | Recent sore throat<br>No recent sore throat |
| | | | sore_throat_1 | N | BF | Had sore throat in last 30 days | | | | | | recent_breathlessness | Bi | Recent shortness of breath / difficulty breathing<br>No recent shortness of breath / difficulty breathing |
| | | | short_breath_diff_1 | N | BF | Had shortness of breath or difficulty breathing in last 30 days | | | | | | recent_headache | Bi | Recent headaches<br>No recent headaches |
| | | | headache_1 | N | BF | Had headache in last 30 days | | | | | | recent_muscle_ache | Bi | Recent muscle ache<br>No recent muscle ache |
| | | | muscle_joint_aches_1 | N | BF | Had general muscle / joint aches / pains in last 30 days | | | | | | recent_fatigue | Bi | Recent fatigue<br>No recent fatigue |
| | | | tiredness_fatigue_1 | N | BF | Has unusual tiredness fatigue / exhaustion in last 30 days | | | | | | recent_sickness | Bi | Recent nausea and / or vomiting<br>No recent nausea and / or vomiting |
| | | | nausea_vomiting_1 | N | BF | Had nausea in last 30 days | | | | | | recent_abdominal_pain | Bi | Recent abdominal pain<br>No recent abdominal pain |
| | | | vomiting_1 | N | BF | Had vomiting in last 30 days | | | | | | recent_diarrhoea | Bi | Recent diarrhoea<br>No recent diarrhoea |
| | | | abdominal_tummy_cramps_1 | N | BF | Has abdominal cramps in last 30 days | | | | | | recent_taste_smell_loss | Bi | Recent loss of taste / smell<br>No recent loss of taste / smell |
| | | | diarrhoea_1 | N | BF | Had diarrhoea in last 30 days | | | | | | | | |
| | | | rash_on_toes_or_feet_1 | N | BF | Had rash on toes or feet in last 30 days | | | | | | | | |
| | | | rash_elsewhere_descr_1 | N | BF | Had rash elsewhere in last 30 days | | | | | | | | |
| | | | loss_of_smell_1 | N | BF | Had loss of smell in last 30 days | | | | | | | | |
| | | | loss_of_taste_1 | N | BF | Had loss of taste in last 30 days | | | | | | | | |
| | | | other_symptoms_1 | N | BF | Other symptoms | | | | | | | | |
| | | | other_rash_location | N | BF | Other rash location | | | | | | | | |
| | Long-COVID symptoms | N/A | | | | | Had symptoms ongoing >4 weeks | end_ongoing_symptoms | S | F | Had persistent COVID-19-related symptoms >4 weeks<br>Had ongoing symptomatic COVID-19<br>Had post-COVID-19 syndrome<br>Had persistent fever<br>Had persistent headache<br>Had persistent muscle ache<br>Had persistent weakness / tiredness<br>Had persistent nausea / vomiting<br>Had persistent abdominal pain<br>Had persistent diarrhoea<br>Had persistent loss of appetite<br>Had persistent loss of taste | long_covid | Bi | Persistent COVID-19-related symptoms >4 weeks<br>No persistent COVID-19-related symptoms >4 weeks |
| | | | | | | | Duration | end_symptoms_duration | S | F | | | | |
| | | | | | | | Symptom ongoing >4 weeks | end_symptoms_SYMPTOM | S | F | | ongoing_symptomatic_covid | Bi | Ongoing symptomatic COVID-19<br>No ongoing symptomatic COVID-19 |
| | | | | | | | | | | | | post_covid_syndrome | Bi | Post-COVID-19 syndrome<br>No post-COVID-19 syndrome |
| | | | | | | | | | | | | persistent_fever | Bi | Persistent fever<br>No persistent fever |

| | | | | | | |
|---|---|---|---|---|---|---|
| | | | Had persistent loss of smell<br>Had persistent sore throat<br>Had persistent cough<br>Had persistent shortness of breath<br>Had persistent chest pain<br>Had persistent palpitations<br>Had persistent vertigo / dizziness<br>Had persistent worry / anxiety<br>Had persistent low mood / not enjoying anything<br>Had persistent trouble sleeping<br>Had persistent memory loss or confusion<br>Had persistent difficulty concentrating<br>Had persistent sensations in skin, such as prickling, tingling or burning<br>Had other persistent symptom | persistent_headache | Bi | Persistent headache<br>No persistent headache |
| | | | | persistent_muscle_ache | Bi | Persistent muscle ache<br>No persistent muscle ache |
| | | | | persistent_fatigue | Bi | Persistent weakness / tiredness<br>No persistent weakness / tiredness |
| | | | | persistent_sickness | Bi | Persistent nausea / vomiting<br>No persistent nausea / vomiting |
| | | | | persistent_abdominal_pain | Bi | Persistent abdominal pain<br>No persistent abdominal pain |
| | | | | persistent_appetite_loss | Bi | Persistent loss of appetite<br>No persistent loss of appetite |
| | | | | persistent_diarrhoea | Bi | Persistent diarrhoea<br>No persistent diarrhoea |
| | | | | persistent_taste_loss | Bi | Persistent loss of taste<br>No persistent loss of taste |
| | | | | persistent_smell_loss | Bi | Persistent loss of smell<br>No persistent loss of smell |
| | | | | persistent_sore_throat | Bi | Persistent sore throat<br>No persistent sore throat |
| | | | | persistent_cough | Bi | Persistent cough<br>No persistent cough |
| | | | | persistent_breathlessness | Bi | Persistent shortness of breath<br>No persistent shortness of breath |
| | | | | persistent_chest_pain | Bi | Persistent chest pain<br>No persistent chest pain |
| | | | | persistent_palpitations | Bi | Persistent palpitations<br>No persistent palpitations |
| | | | | persistent_anxiety | Bi | Persistent worry / anxiety<br>No persistent worry / anxiety |
| | | | | persistent_dizziness | Bi | Persistent vertigo / dizziness<br>No persistent vertigo / dizziness |
| | | | | persistent_depression | Bi | Persistent low mood / not enjoying anything<br>No persistent low mood / not enjoying anything |
| | | | | persistent_insomnia | Bi | Persistent trouble sleeping<br>No persistent trouble sleeping |
| | | | | persistent_memory_loss | Bi | Persistent memory loss or confusion<br>No persistent memory loss or confusion |
| | | | | persistent_difficulty_concentrating | Bi | Persistent difficulty concentrating<br>No persistent difficulty concentrating |
| | | | | persistent_skin_sensations | Bi | Persistent sensations in skin, such as prickling, tingling or burning<br>No persistent sensations in skin, such as prickling, tingling or burning |
| | | | | persistent_other | Bi | Persistent other symptom<br>No persistent other symptom |

| | | | | | | | | | | | | | | |
|---|---|---|---|---|---|---|---|---|---|---|---|---|---|---|
| Educational | School absences | No. days off school due to non-COVID-19 illness in past 30 days | symp_no_absnt_30_days | N | BF | Off school due to COVID-19-related reason in past 30 days<br>Off school due to illness not related to COVID-19 in past 30 days<br>No. days off school due to medically diagnosed COVID-19 (confirmed by a doctor) in past 30 days<br>No. days off school due to suspected COVID-19 (not medically confirmed) in past 30 days<br>No. days off school because of other illness not related to COVID-19 in past 30 days | Absent in past 4 weeks for any reason | fw_absent | S | F | Off school due to COVID-19-related illness in past 28 days<br>Off school due to illness not related to COVID-19 in past 28 days<br>No. days off school due to any illness in past 28 days if all absences due to illness | off_school_illness | Bi | Off school due to either COVID-19 or non-COVID-19 illness in past month<br>Not off school due to either COVID-19 or non-COVID-19 illness in past month |
| | | No. days off school due to COVID-19 illness in past 30 days | symp_no_covid_absnt_30_days | N | BF | | No. days off school missed in past 4 weeks for any reason | fw_absent_no | S | F | | no_days_off_school_illness | N | No. days off school due to COVID-19 or non-COVID-19 illness in past month |
| | | No. days off school due to confirmed COVID-19 in past 30 days | symp_no_covid_absnt_confirmed_by_dr | N | BF | | Any absences COVID-19 related | fw_absent_reason | S | F | | | | |
| | | No. days off school due to unconfirmed COVID-19 in past 30 days | symp_no_suspected_covid_absnt_30_days | N | BF | | COVID-19 related reason(s) | fw_absent_not_covid | S | F | | | | |
| | | No. days off school due to non-COVID-19 illness in past 30 days | symp_no_other_absnt_30_days | N | BF | | Non-COVID-19 related reason(s) | fw_absent_covid | S | F | | | | |

N – number; T – free text; S – string; D – datetime; Bi – binary; C – categorical; B – baseline; F – follow up; BF – baseline and follow up.
