# Supplementary material for: Acute and long-term outcomes of SARS-CoV-2 infection in school-aged children in England: Study protocol for the joint analysis of the COVID-19 schools infection survey (SIS) and the COVID-19 mapping and mitigation in schools (CoMMinS) study
Source: PLoS One. 2024 May 22;19(5):e0303892. doi: 10.1371/journal.pone.0303892 (PMC11111005; doi:10.1371/journal.pone.0303892)
Supplement: S2 Table — (PDF) [file pone.0303892.s003.pdf]

S2 Table: Analysis risk factors (RQ1)/covariates (RQ2), description and informing data sources.

Risk factor/Covariate	Type	Categories	Data sources
Gender	Categorical	Male, Female, Other	SIS-1/CoMMinS data, or EHR if missing
Age	Continuous	Age in years	SIS-1/CoMMinS data, or EHR if missing
Ethnicity	Categorical	Black/African/Caribbean/Black British; Asian/Asian British; White; Other ethnic group; Mixed/Multiple ethnic groups	SIS-1/CoMMinS data, or EHR if missing
Deprivation	Categorical	Index of Multiple Deprivation 2019	ONS (from SIS-1 data)/CoMMinS data
Symptomatic SARS-CoV-2 infection (RQ1 only)	Binary	Yes, No	SIS-1/CoMMinS data
SARS-CoV-2 vaccination	Datetime	Date of first vaccination	SIS-1/CoMMinS and EHR data
School type	Categorical	Primary, Secondary, Sixth Form	SIS-1/CoMMinS data
Geographical location	Categorical	Middle Layer Super Output Area (MSOA), local authority district, integrated care system	ONS (from SIS-1 data)/CoMMinS data
Asthma	Binary	Yes, No	SIS-1/CoMMinS and EHR data
Diabetes	Binary	Yes, No	SIS-1/CoMMinS and EHR data
Heart condition	Binary	Yes, No	SIS-1/CoMMinS and EHR data
Other long-term medical condition	Binary	Yes, No	SIS-1/CoMMinS and EHR data
Hospital admission in the previous 12 months	Binary	Yes, No	EHR data
SARS-CoV-2 variant	Categorical	S-gene status in SIS, time of infection in CoMMinS	SIS-1/CoMMinS data
