# Supplementary material for: Acute and long-term outcomes of SARS-CoV-2 infection in school-aged children in England: Study protocol for the joint analysis of the COVID-19 schools infection survey (SIS) and the COVID-19 mapping and mitigation in schools (CoMMinS) study
Source: PLoS One. 2024 May 22;19(5):e0303892. doi: 10.1371/journal.pone.0303892 (PMC11111005; doi:10.1371/journal.pone.0303892)
Supplement: S3 Table — (PDF) [file pone.0303892.s004.pdf]

S3 Table: Definition of ongoing symptomatic COVID-19 and post-COVID-19 syndrome using self-reported symptom data from CoMMinS versus SIS.

	Persistent self-reported symptoms	
	**4-12 weeks**	**>12 weeks**
**CoMMinS**	Symptom reported as experienced during past 30 days in two or more survey rounds with 4-12 weeks of each other	Symptom reported as experienced during past 30 days in two or more survey rounds, with the first and last report >12 weeks of each other
**SIS**	(a) Symptom reported as experienced during +/- 7 days of sampling in two or more survey rounds with 4-12 weeks of each other<br>(b) Symptom reported as ongoing for 4-12 weeks	(a) Symptom reported as experienced during +/- 7 days of sampling in two or more survey rounds, with the first and last report >12 weeks of each other<br>(b) Symptom reported as ongoing for >12 weeks
