# Supplementary material for: Acute and long-term outcomes of SARS-CoV-2 infection in school-aged children in England: Study protocol for the joint analysis of the COVID-19 schools infection survey (SIS) and the COVID-19 mapping and mitigation in schools (CoMMinS) study
Source: PLoS One. 2024 May 22;19(5):e0303892. doi: 10.1371/journal.pone.0303892 (PMC11111005; doi:10.1371/journal.pone.0303892)
Supplement: S4 Table — (PDF) [file pone.0303892.s005.pdf]

S4 Table: Expected challenges/limitations, and steps that will be taken to minimise/mitigate effect.

Challenge/Limitation	What will be done to minimise/mitigate effect
**Loss-to-follow-up**<br>Some infections and symptoms may be missed where participants are not surveyed at a given study round	• Inclusion of survey testing, primary care and Second Generation Surveillance System data to increase the chances of identifying all active infections<br>• Inclusion of antibody data to identify infections not identified through testing for active infection
**Surveys may not adequately capture associated morbidity**<br>Symptom list is not exhaustive, and symptoms do not have an associated definition	• Triangulation of long-term effect of SARS-CoV-2 infection from multiple sources<br>• Survey list for both SIS and CoMMinS includes those symptoms considered most harmful in PPI work(1)
**Evolving nature of the pandemic**<br>SIS and CoMMinS are historic studies that took place when the wild-type and delta-type SARS-CoV-2 variants were circulating	• It is importance to understand the past dynamics of SARS-CoV-2<br>• The health effects of earlier variants continue to be felt
**Misclassification bias**<br>Asymptomatic infections may be less likely to be identified than symptomatic infections (outside of testing done through the surveys) as a consequence of government testing strategy, potentially leading to either an under- or overestimation of outcome risk associated with SARS-CoV-2 infection	• Inclusion of survey testing to increase the chances of identifying all active infections<br>• Inclusion of antibody data to identify asymptomatic infections/infections not identified through testing for active infection
**Selection bias**<br>Those at risk of developing long-COVID (e.g., potentially those with symptomatic acute SARS-CoV-2 infection, or those with pre-existing health conditions) may be more likely to participate in the surveys	• Adjustment of effect estimates for covariates<br>• Subgroup analyses by participant demographic and clinical characteristics<br>• Inclusion of antibody data to identify asymptomatic infections/infections not identified through testing for active infection
**Response bias**<br>Participants with SARS-CoV-2 infection may overreport symptoms. Survey participants are not presented with symptom definitions in either CoMMinS or SIS	• Inclusion of antibody data to identify those with an unrecognised SARS-CoV-2 infection<br>• Triangulation of long-term effect of SARS-CoV-2 infection from multiple sources
**Inadequate study power**<br>Outcome categories may be too broad and/or numbers too small to detect associations	• Use of two different cohorts to investigate associations<br>• Combining outcomes may conversely increase the power to detect an association
**Chance associations**<br>At (e.g.) the 5% level (i.e., using $p<0.05$ as a cut-off which is a less than 5% chance that the null hypothesis of no association is false), 1 in 20 associations investigated will be statistically-significant purely on the basis of chance	• ICD-10 and BNFC chapters selection informed by clinical consultation, documented symptomatic SARS-COV-2 in children, how well they are reported, and PPI work<br>• Evidence across multiple outcome types will help determine plausibility of associations (e.g., comparing associations across relevant related survey symptoms, diagnoses and prescriptions for a given condition)
**Missing data**<br>Missing data on socio-demographic and clinical covariates may bias, or reduce the power to detect, effect estimates, if missing data are not random (i.e., more likely for certain covariate categories) and confound/modify the association between exposure and outcome	• Use of both research data and EHR data to inform covariates<br>• Exploration of patterns in missing data and use of multiple imputation where appropriate
**Incomplete outcome reporting in EHRs**<br>Not all outcomes recorded due to incomplete or variable recording by healthcare professionals, and individuals not presenting to healthcare services	• Association of SARS-CoV-2 infection with multiple different types of outcomes investigated to increase likelihood of capturing effect of infection on long-term health outcomes
**Use of data on recent symptoms as a proxy for persistent symptoms**<br>A symptom being reported as experienced in multiple survey rounds does not necessarily mean the symptom was persistent the whole time. SIS and CoMMinS do not define 'recent' symptoms in the same way	• The assumption that repeated symptoms correlate with persistent symptoms is not necessarily an issue if long-COVID symptoms are fluctuating<br>• Persistent symptoms will be analysed in two ways for SIS, with use of data collected specifically on persistent symptoms as well as repeated data collection on recent symptoms
**Outcomes due to other causes**<br>The outcomes investigated (symptoms, school absences due to illness, diagnoses of conditions, medication use and health service attendance) have multiple other potential causes/explanations	• Use of a control/unexposed group<br>• We are utilising all available data, and comparing hazard ratios for time since SARS-CoV-2 diagnosis, with follow up without or before infection for each outcome<br>• Adjustment of effect estimates for covariates

	• Sensitivity analyses
**Differential clinical course in different individuals**<br>The long-term effects of SARS-CoV-2 infection may be seen in different CYP in different ways	• Use of broad chapters of diagnoses and prescriptions to more fully capture all possible long-term effects of SARS-CoV-2 infection<br>• Clustering and latent class analyses planned
